# Expression and Clinical Significance of *MAPK8*, *MAPK9*, *MAP2K4*, and *MAP2K7* Genes in Colorectal Cancer

**DOI:** 10.3390/ijms27010100

**Published:** 2025-12-22

**Authors:** Agnieszka Wosiak, Damian Wodziński, Rafał Świechowski, Jacek Pietrzak, Michał Mik, Ewa Balcerczak

**Affiliations:** 1 Department of Pharmaceutical Biochemistry and Molecular Diagnostics, Medical University of Lodz, Muszynskiego 1, 90-151 Lodz, Poland; 2 BRaIn Laboratories, Medical University of Lodz, Czechoslowacka 4, 92-216 Lodz, Poland; 3 Department of General and Colorectal Surgery, Medical University of Lodz, ul. Zeromskiego 113, 90-549 Lodz, Poland

**Keywords:** colorectal cancer, MAPK signalling, PCR, MAPK8, MAPK9, MAP2K4, MAP2K7, JNK signalling pathway

## Abstract

This study investigated whether alterations in the expression of genes integral to the c-Jun N-terminal kinase (JNK) signaling pathway play a role in the pathogenesis of colorectal cancer (CRC). It analyzed the expression of genes encoding two JNK isoforms (MAPK8 and MAPK9) and the JNK-activating kinases (MAP2K4 and MAP2K7). Gene expression patterns in CRC tissue were compared with existing data in public online databases to provide an integrated understanding of their potential role in tumorigenesis. The material consisted of 55 cancer tissue fragments collected intraoperatively from patients with histopathologically confirmed CRC. Total RNA isolated from these tissues was used to determine the relative expression of the selected genes using quantitative PCR. Additionally, data from publicly accessible bioinformatics databases were utilized. *MAP2K7* gene expression was significantly elevated in tumor specimens with higher histological grades. Conversely, *MAPK9* gene expression tended to be higher in tumor tissues with lower histological grades. Moreover, elevated MAPK8 gene expression was linked to an increased incidence of regional lymph node metastasis. Furthermore, bioinformatics analysis confirmed that *MAP2K7* and *MAPK8* appear to promote tumor aggressiveness and metastasis, whereas *MAPK9* and *MAP2K4* may have a protective or regulatory role in early stages of the disease.

## 1. Introduction

Colorectal cancer (CRC) is the second most prevalent malignancy worldwide. While the greatest prevalence has been observed among individuals over the age of 50, recent statistics indicate a rising incidence among younger demographics. Epidemiologically, colorectal carcinogenesis is a multifaceted and sequential process characterized by aberrations in the proliferation, apoptosis, and differentiation of colonic epithelial cells, modulated by an interplay of epidemiological, environmental, and genetic determinants [1]. According to the World Health Organization (WHO), in 2020, CRC accounted for approximately 1.93 million new cases and 935,000 deaths globally, underscoring its significant health burden. Furthermore, it has been found that genetic predispositions contribute to roughly 35% of CRC cases, highlighting the critical role of hereditary factors in its pathogenesis. The risk is further compounded by environmental influences, such as diet, physical inactivity, and smoking, demonstrating the complex etiology of this disease [2]. Therefore, further investigation into CRC pathogenesis is needed to improve disease understanding, refine diagnostic approaches, and develop more effective preventive and therapeutic interventions [3].

In CRC, mutation or gene overexpression can result in dysregulation of the mitogen-activated serine–threonine kinase (MAPK) pathway [4]. This pathway includes several key components, such as RAF, MEK, and ERK. Mutations in genes like *KRAS* and *BRAF* are particularly common in CRC and can lead to constitutive activation of the MAPK pathway. Activation by *KRAS* can result in an imbalance between ERK and JNK activity, and this could play a crucial role in human colorectal carcinogenesis [5,6]. Aberrant activation of JNK may increase cell proliferation and inhibit apoptosis, supporting the survival of damaged or mutated cells and allowing abnormal cells to accumulate and form tumors in the colon. While DNA damage typically leads to the activation of the JNK pathway, directing the cell to the apoptosis pathway, it may contribute to the accumulation of mutations in cancerous cells and accelerate cancer progression. The JNK pathway can also promote angiogenesis, and it may be involved in inducing resistance to chemotherapy and targeted therapy in intestinal cancer cells and may contribute to reduced treatment effectiveness [7].

The aim of this investigation was to quantitatively evaluate the expression profiles of genes implicated in the biosynthesis of proteins that activate the MKK4/7-JNK signaling cascade (specifically *MAP2K4* and *MAP2K7*), as well as genes coding for two isoforms of the JNK enzyme (*MAPK8* and *MAPK9*). Bioinformatic tools were also used to assess the relationship between the expression of the studied genes and factors such as overall patient survival, the molecular status of genes implicated in CRC development, and the tumor’s molecular subtype.

Published data indicate that the genes selected for analysis are involved in key cellular processes, and available manuscripts support an association between alterations in the expression profile of genes belonging to the MAPK pathway and cancer development [4,6]. A more detailed understanding of the cellular mechanisms involving the JNK pathway may support the development of therapies targeting components of this signaling cascade and the advancement of novel diagnostic methods.

## 2. Results

The expression of the studied genes was compared with the demographic characteristics of patients with CRC, including age and sex. No significant correlation was found between the level of gene expression and either age or sex.

**Figure 1:**
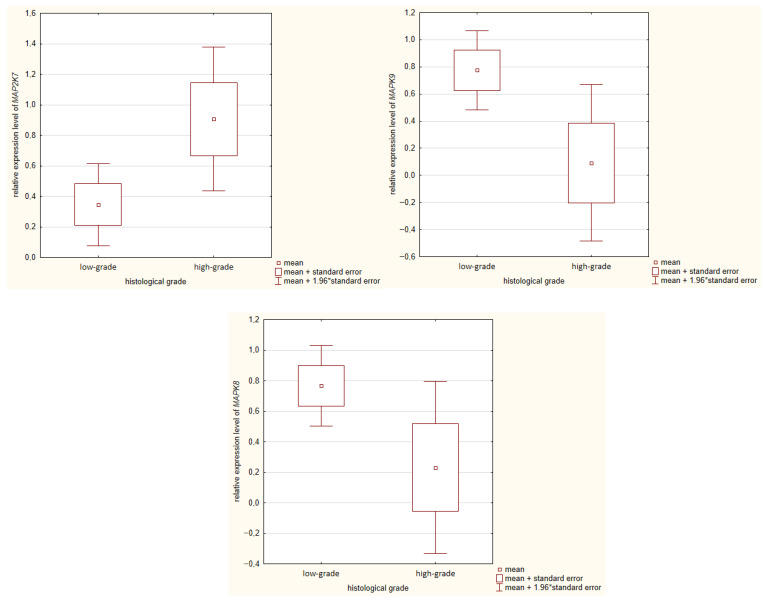
Comparison of MAP2K7, MAPK9, and MAPK8 gene expression and histological grade of CRC. * multiplication sign.

A significant association was found between *MAP2K7* and *MAPK9* expression and the histological grade of colorectal tumors. Elevated *MAP2K7* gene expression was significantly correlated with high histological grade (G3–G4) (*p* = 0.042), while increased *MAPK9* expression was significantly associated with lower histological grade (G1–G2) (*p* = 0.029). Additionally, elevated *MAPK8* expression was observed in tumors graded G1/G2, although this was of borderline significance (*p* = 0.063). These results are illustrated in Figure 1.

**Figure 2:**
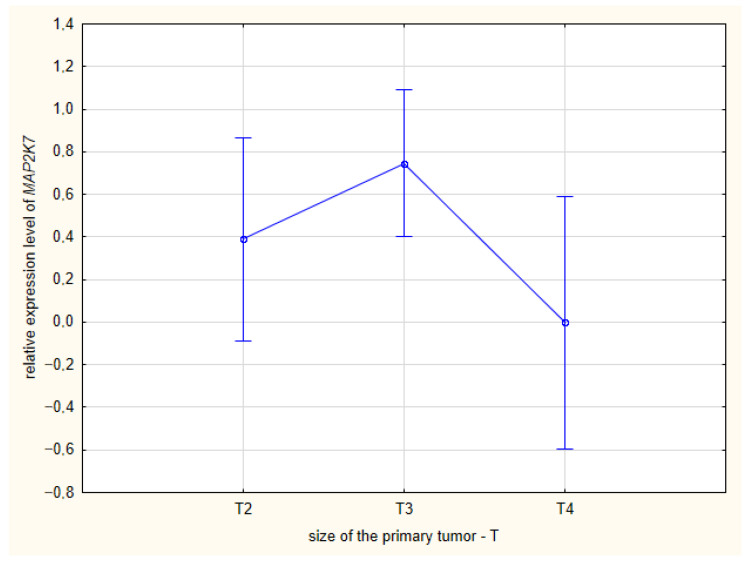
Comparison of *MAP2K7* gene expression and primary tumor size.

The expression of the tested genes was also compared with the size of the primary tumor (T) according to the TNM classification. An insignificant tendency toward higher *MAP2K7* expression was observed in primary tumors with lower infiltration (T3) compared those rated as T4 *(p* = 0.0852). The results are shown in Figure 2.

**Figure 3:**
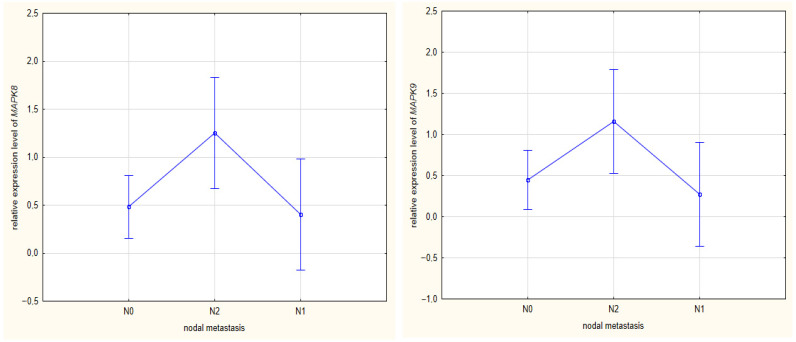
Comparison between *MAPK9* and *MAPK8* expression and the nodal metastasis of CRC.

Regarding the presence of metastases to regional lymph nodes (N) according to the TNM classification, significantly higher *MAPK8* expression was noted in patients with metastases to at least four lymph nodes compared to those showing no metastases (N0) (*p* = 0.0239) or only one lymph node (N1) (*p* = 0.042). A similar statistical tendency was demonstrated for the *MAPK9* gene: N0 *p* = 0.055 and N1 *p* = 0.052. The results are presented in Figure 3.

No significant relationship was found between gene expression and clinicopathological features including tumor location, cancer stage according to TNM classification, presence of distant metastases, and presence of neuroinvasion or angioinvasion.

### Bioinformatics Analysis

**Figure 4:**
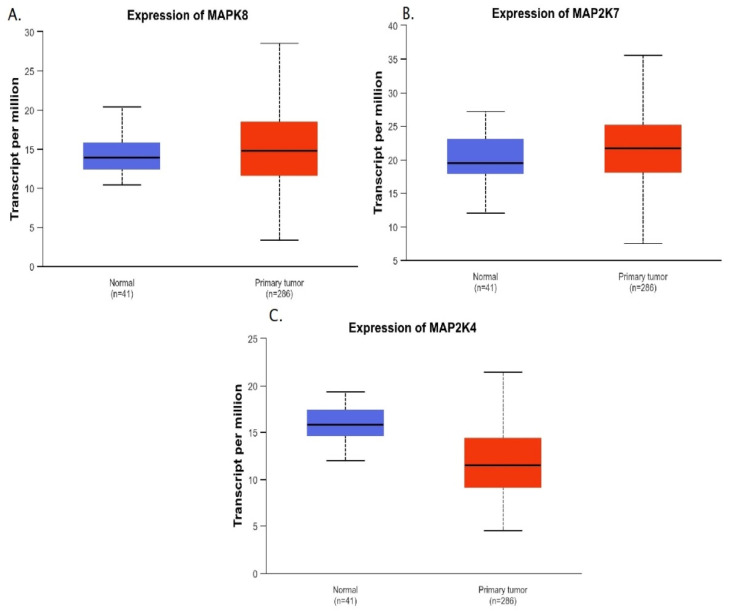
Comparison of *MAPK8*, *MAP2K7*, and *MAP2K4* gene expression between colon tumor tissue and normal colon tissue.

First, gene expression was compared between normal colon tissues and cancer tissues. Significantly higher levels of *MAPK8* and *MAP2K7* gene expression were observed in cancerous tissues (*p* = 0.0466; *p* = 0.0478), while higher *MAP2K4* expression was observed in healthy colon tissues (*p* < 0.0001) (Figure 4).

**Figure 5:**
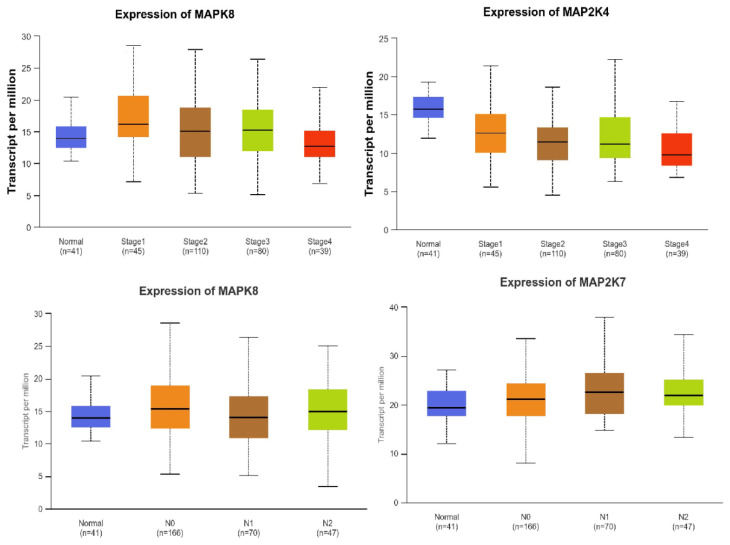
Comparison of *MAPK8*, *MAP2K4*, and *MAP2K7* gene expression with various clinicopathological features.

Higher levels of *MAPK8* and *MAP2K4* gene expression were most frequently correlated with the lowest stage of cancer (stage 1 compared to stage 4) (*p* = 0.0036; *p* = 0.0339). Higher levels of *MAPK8* and *MAP2K7* gene expression were observed in patients with lymph node metastases (number 1–3) compared to patients without lymph node involvement (*p* = 0.0382; *p* = 0.0079). The results of these analyses obtained from the UALCAN database are presented in Figure 5.

**Figure 6:**
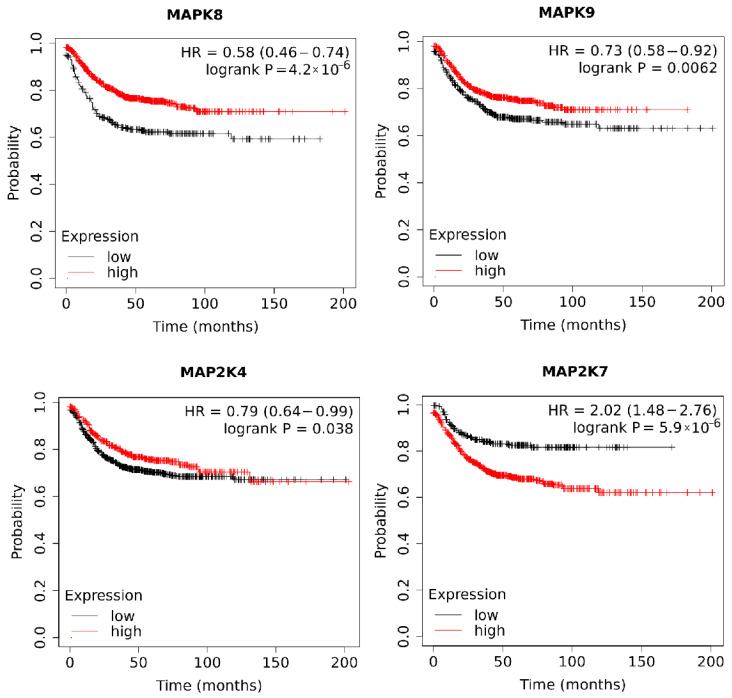
Kaplan–Meier curves representing the survival rate of patients with colorectal cancer in relation to *MAPK8*, *MAPK9*, *MAP2K4*, and *MAP2K7* expression.

Regarding the impact of *MAPK8*, *MAPK9*, *MAP2K4*, and *MAP2K7* expression on overall survival (OS), significantly longer survival times were observed in patients with higher expression of *MAPK8* (*p* < 0.0001), *MAPK9* (*p* = 0.0062), and *MAP2K4* (*p* = 0.038). However, lower *MAP2K7* expression correlated with longer patient survival (*p* < 0.0001). The results are presented in Figure 6.

**Figure 7:**
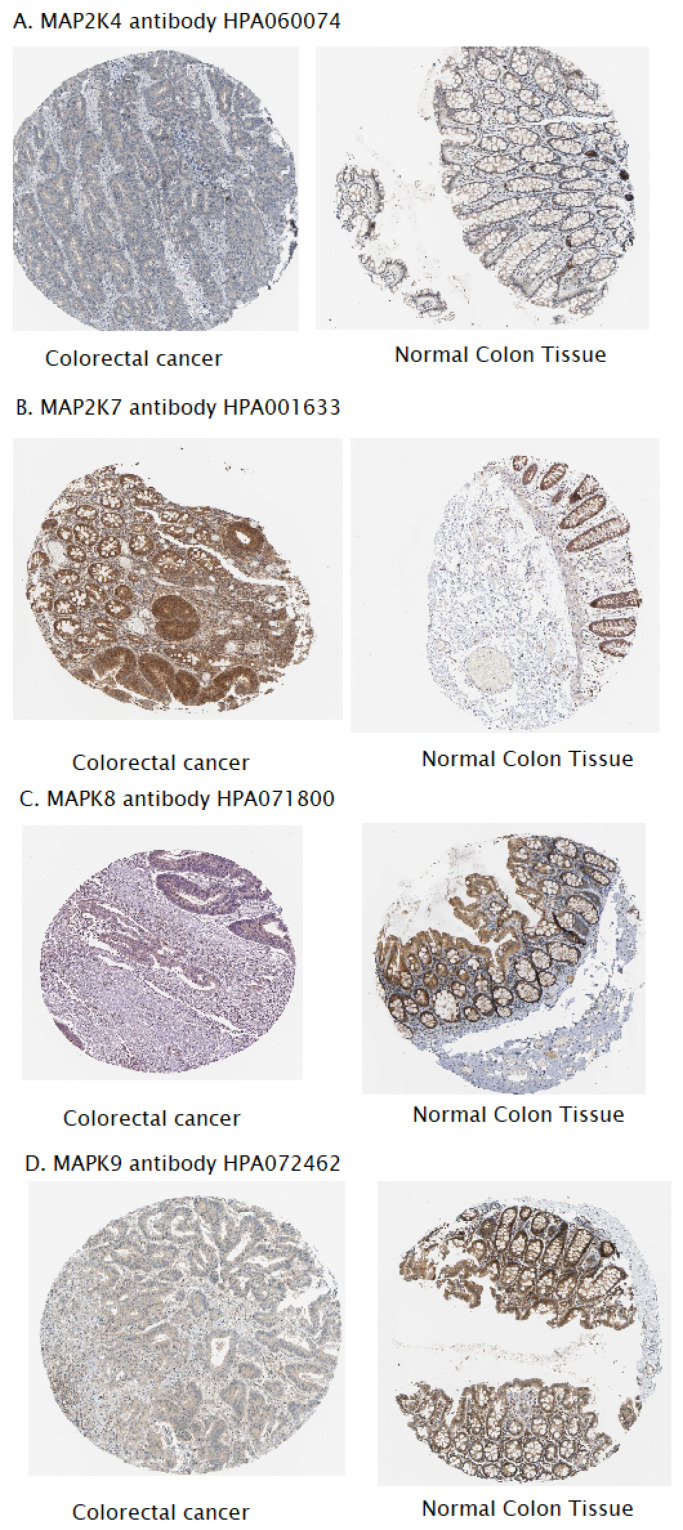
Comparison of protein expression levels in colon cancer tissue and normal colon tissue (Human Protein Atlas).

After assessing the mRNA expression levels of selected genes, we assessed the expression of MAP2K4, MAP2K7, MAPK8, and MAPK9 proteins in cancer and normal colon tissue using images from the Human Protein Atlas. MAP2K4 protein expression was undetectable in both colon tissues. However, we found that high levels of MAP2K7 protein expression were observed in colon cancer tissue, while moderate expression was observed in normal tissue. No or low expression of MAPK8 protein was detected in colon cancer tissue, while moderate expression was observed in normal tissue. Moderate expression of MAPK9 protein was observed in cancer tissue, while high expression was observed in normal tissue. The results are presented in Figure 7.

The association between *MAP2K4*, *MAP2K7*, *MAPK8*, and *MAPK9* gene expression and the presence of mutations in the *KRAS*, *PIK3CA*, *BRAF*, and *TGFB1* genes was assessed using the TIMER 2.0 platform. The analysis included data from 401 colorectal adenocarcinoma (COAD) and 144 rectal adenocarcinoma (READ) samples. The analysis revealed an association between higher levels of *MAP2K4* and *MAPK8* gene expression and the presence of *BRAF* mutations in COAD samples. Similarly, higher levels of *MAP2K4* gene expression were observed in COAD samples in the presence of the *TGFB1* gene. The results are presented in Table 1.

**Figure 8:**
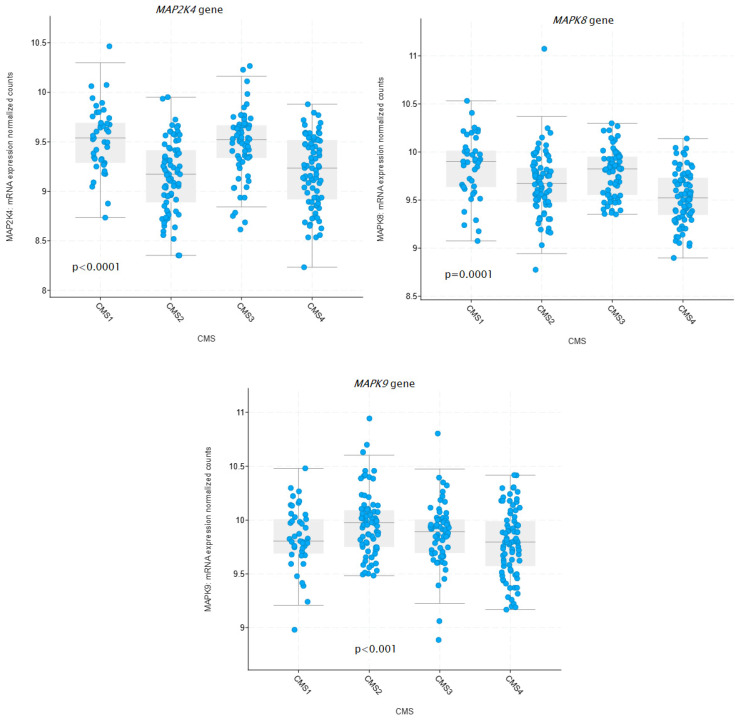
Differences in the expression of MAPK8, MAPK9, MAP2K4, and MAP2K7 genes in colorectal cancer tissue according to CMS. *p*-values were calculated using one-way ANOVA.

The analysis of the association between the expression levels of the *MAP2K4*, *MAP2K7*, *MAPK8*, and *MAPK9* genes and the molecular subtype of CMS colorectal cancer was performed using data available at https://www.cbioportal.org/ accessed on 6 May 2025. Data from 267 CRC patients, classified into CMS groups, were used for the analysis. A statistically significant association was demonstrated between higher expression levels of the *MAP2K4* and *MAPK8* genes in the CMS1 subtype (*p* < 0.0001) and higher expression levels of the *MAPK9* gene in the CMS2 subtype (*p* < 0.001). The results are presented in Figure 8.

## 3. Discussion

Approximately 50% of CRC-harboring mutations in the *KRAS* gene exhibit hyper activation of the MAPK signaling pathway, potentially accelerating tumor progression. Genes within the MAPK cascade are essential for regulating the proliferation and differentiation of colonic epithelial cells, and its dysregulation can frequently stimulate the oncogenesis of CRC [8].

The aim of this study was to determine the role of genes acting as key mediators of the MAPK pathway in the development of CRC. Our analysis on a group of patients diagnosed with CRC suggests that these genes may in fact play a part in the course of cancer. Several connections were found between the expression of *MAPK* pathway genes and certain pathological parameters associated with CRC. Interestingly, different correlations were found for each gene. However, due to the complex nature of intestinal carcinogenesis and the influence of numerous factors, many of which were not assessed in the present study, the obtained results remain difficult to interpret.

Higher expression of *MAP2K7* was found in higher-grade tumors (G3/G4), which may suggest that *MAP2K7* plays a role in more advanced stages of CRC. *MAP2K7* is known to activate JNKs, which may promote proliferation and resistance to cellular stress in cancer cells. Furthermore, the results regarding patient survival suggest that lower expression of *MAP2K7* is associated with longer survival, which may confirm its role in tumor progression [9].

The higher expression of *MAPK9* observed in lower-grade tumors (G1/G2) may indicate a protective or adaptive role of *MAPK9* in the early stages of cancer. *MAPK9* encodes one of the JNK isoforms, which may exhibit different functions depending on the development of the tumor—for example, in some cases, JNK activation may lead to apoptosis of tumor cells, while in others it may lead to cell survival [10].

Increased expression of *MAPK8* was correlated with the presence of lymph node metastasis. *MAPK8* (encoding JNK1) may play a key role in promoting the invasive and migratory potential of tumor cells.

*MAP2K4* is often described as a tumor suppressor gene that may act as a brake on cell proliferation. This is supported by the observed lower expression of *MAP2K4* in tumor tissues compared to healthy tissues. Its reduced expression in tumor tissue may indicate a loss of this protective function, allowing for rapid tumor development [11].

The results suggest that the JNK pathway plays a complex role in CRC, and individual genes can act as both promoters and inhibitors of tumor progression, depending on the histopathological type and stage of the tumor. *MAP2K7* and *MAPK8* may promote disease progression and metastasis, whereas *MAPK9* and *MAP2K4* may be more protective at certain stages of cancer development. Our findings demonstrate that differential expression of MAPK pathway genes correlates with tumor development, highlighting potential biomarkers for prognosis and targets for therapeutic intervention in CRC.

An in vitro study by Cristina Blaj et al. investigating the relationship between MAPK pathway activity and cancer cell growth rate found that CRC cell cultures with high MAPK activity were characterized by loss of E-cadherin and exhibited mesenchymal–epithelial transition. Moreover, they confirmed that the activation of the MAPK pathway correlates with the activation of the WNT pathway, which promotes the proliferation of cancer cells [12]. Similarly, Jingzhi Tang et al. report that activation of the MAPK pathway in CRC cells in vivo promotes cell proliferation and regulates cell cycle and apoptosis of human colon cancer [13].

Baraul et al. reported that increased activation of the MAPK pathway may be related to shorter survival of patients with colorectal cancer. Activation of the RAS-MAPK, PI(3)K cascade through the occurrence of point mutations in *KRAS*, *PIK3CA*, or *BRAF* genes may be associated with worse patient survival and worse response to chemotherapy drugs [14]. Upregulation of the MAPK cascade is also common in other types of cancers, for example, hepatocellular carcinoma (HCC) [15].

A study on the role of miRNA molecules in regulating the *MAP2K4* suppressor gene found that inhibition of its expression promotes the proliferation of colon cancer cells. A lower level of gene expression was noted in CRC cells from different cell lines compared to normal colorectal cells [16]. Similar results were obtained by analyzing data from the UALCAN database. Reduced *MAP2K4* gene expression results in decreased production of the kinase needed by the MAPK signaling pathway to stimulate apoptosis and hence a poor prognosis in colorectal cancer. The key role played by *MAP2K4* gene expression in the development and progression of CRC may serve as a potential therapeutic target for drugs used in chemotherapy for patients with advanced CRC.

It was found that the downregulation of *MAP2K4* gene expression by methylation is closely associated with a worse prognosis in CRC. Patients with CRC exhibited reduced activity of the protein encoded by the *MAP2K4* gene compared to healthy individuals; moreover, this loss of activity correlated with the occurrence of cancer metastases to lymph nodes and distant organs and with the clinical stage of the primary cancer. Furthermore, higher level of MKK4 activity in these patients was associated with longer OS [12]. This correlation was confirmed using the Kaplan–Meier plotter for survival analysis based on information available in the database. These results support the role of the *MAP2K4* gene as a tumor suppressor in the development of colorectal cancer. However, in the present study, no relationship was found between the level of *MAP2K4* expression and the occurrence of specific pathological parameters related to the progression of CRC, although this may be due to the use of a small study group.

The prognosis of patients with advanced CRC is influenced by the presence of liver metastases. Sakai et al. confirm that *MAP2K7* gene expression is required to activate the phosphorylation of JNK proteins, which is necessary for the proliferation of CRC cells. The results indicate a relationship between increased *MAP2K7* gene expression and the occurrence of cancer metastases in the liver in patients with CRC [17]. Our present findings also indicate that the level of gene expression correlates with the degree of histological malignancy of the tumor, and our survival analysis confirms that lower *MAP2K7* gene expression is associated with longer survival among CRC patients. Future therapies based on inhibiting the expression of selected genes may reduce the risk of metastasis in patients with CRC.

Bioinformatic analyses further confirm the complex role of JNK pathway genes in colorectal carcinogenesis. Data from the Human Protein Atlas revealed differential protein expression of MAP2K4, MAP2K7, MAPK8, and MAPK9 between tumor and normal colon tissues; notably, MAPK8 protein expression did not correspond to the mRNA-level findings. These differences may result from the simple fact that both observations come from completely different sample sets. Among equally probable causes of such a phenomenon, we can include post-translational modifications, which may alter the stability of the protein and its accessibility to the antibodies used, as well as protein degradation processes occurring in the tumor microenvironment.

The high MAP2K7 protein expression observed in CRC tissues may promote JNK phosphorylation and activation, supporting tumor progression, as also indicated by previous studies on MAP2K7-mediated oncogenic signaling [9,18]. Conversely, the reduced expression of the MAP2K4 protein in tumor tissues reinforces its recognized function as a tumor suppressor, consistent with earlier findings in ovarian cancer [11]. Data obtained from the TIMER 2.0 platform, which integrates gene expression with mutational profiles, revealed significant associations between increased expression of *MAP2K4* and *MAPK8* and the presence of *BRAF* mutations, as well as between *MAP2K4* expression and *TGFB1* mutations in CRC. These findings suggest that dysregulation of the MAPK–JNK axis may coexist with alterations in these genes, constituting an alternative pathway for colorectal cancer progression [18]. Furthermore, molecular subtyping analysis using cBioPortal revealed that *MAP2K4* and *MAPK8* expression levels were significantly elevated in the CMS1 subtype, which is characterized by strong immune infiltration in the tumor microenvironment and high microsatellite instability and frequent mutations in the *BRAF*, *PTEN*, and *ATM* genes. This association is biologically plausible, as both MAP2K4 and MAPK8 are key regulators of stress-induced and proinflammatory signaling. CMS1 tumors demonstrate marked activation of immune-related transcriptional pathways, including interferon- and cytokine-driven pathways known to interact with the MAPK-JNK axis. MAP2K4 is involved in the activation of JNK and p38 kinases in response to genomic instability and inflammatory stimuli—features strongly enhanced in MSI-H tumors. Similarly, MAPK8 participates in cytokine signaling and T-cell-dependent inflammatory responses, consistent with the immune-rich microenvironment characteristics of CMS1 tumors. Together, these patterns suggest that elevated MAP2K4 and MAPK8 expression may reflect the intense inflammatory and stress conditions typical of CMS1 tumors [19]. In contrast, higher *MAPK9* expression was observed in the CMS2 subtype, associated with chromosomal instability, *APC* mutations, and activation of the Wnt and Myc pathways [20]. This pattern suggests that different components of the MAPK cascade may differentially modulate the tumor microenvironment and immune response, highlighting their potential role as subtype-specific therapeutic targets. MAPK9 has been shown to promote tumor growth by stabilizing MYC and facilitating proliferative signaling—key features of the CMS2 subtype. Unlike MAPK8, which can promote apoptosis in certain contexts, MAPK9 is more often associated with pro-survival and pro-proliferative functions. Elevated MAPK9 expression in CMS2 is therefore consistent with strong activation of growth-promoting pathways in this subtype. Furthermore, CIN-positive tumors exhibit increased mitotic and replicative stress, which may further induce MAPK9-dependent compensatory survival signaling [21].

## 4. Materials and Methods

### 4.1. Material

The study group comprised 55 patients from the Department of General and Colorectal Surgery of the Medical University in Lodz, Poland; all were diagnosed with CRC based on histopathological examination. The biological material for the study consisted of CRC tissues collected intraoperatively. Tumor tissues were classified according to tumor histological grade based on the WHO classification system as follows: G1 (well-differentiated), G2 (moderately differentiated), G3 (poorly differentiated), and G4 (undifferentiated). The group of patients included 28 women and 27 men, and the median age of the participants was 69.5 years (minimum 34 years, maximum 90 years). The clinical characteristics of the study group are presented in Table 2. The research on tissues was performed with the consent of the Bioethics Committee of the Medical University of Lodz (approval number RNN/84/20/KE∔03.03.2020 r. with amendments numbered KE/951/22—11.10.2022 r., and KE/92/24—13.02.2024 r.).

### 4.2. Methods

#### 4.2.1. RNA Isolation

RNA was isolated from tumor tissues obtained intraoperatively from patients diagnosed with CRC. Immediately after collection, the tissues were placed in StayRNA™ stabilization solution (A&A Biotechnology, Gdansk, Poland) and stored at −80 °C. The RNA isolation procedure was performed using the RNeasy Mini Kit (Qiagen, Hilden, Germany) in accordance with the manufacturer’s recommendations. The concentration and purity of the obtained RNA were assessed spectrophotometrically using a NanoPhotometer (Implen, München, Germany). RNA samples with an A260/A280 nm absorbance ratio of 1.8 to 2.0 were considered pure and used for analyses.

#### 4.2.2. Reverse Transcription Reaction

Total RNA was reverse transcribed into cDNA using the High-Capacity cDNA Reverse Transcription Kit (Applied Biosystems, San Francisco, CA, USA) according to the manufacturer’s attached protocol. In the first step, a reaction mixture containing 10 × RT Buffer, Oligo(dT)18 Primer, 25 × dNTP Mix (100 mM), MultiScribe™ Reverse Transcriptase (50 U/μL), and RNase Inhibitor was prepared. All samples were made up to 0.03 µg/µL before analysis by adding an appropriate amount of distilled water. The reverse transcription reaction itself was performed using the same RNA concentration for all samples. Each reaction was carried out under the following conditions: 10 min at 25 °C, then 120 min at 37 °C and 5 min at 85 °C. The obtained cDNA samples were stored at −20 °C until further analyses.

#### 4.2.3. Real-Time PCR

The expression of each tested gene (*MAPK8*, *MAPK9*, *MAP2K4*, *MAP2K7*) relative to reference genes (*GAPDH*, *ACTB*) was determined by quantitative real-time PCR using specific TaqMan™ molecular probes (Thermo Fisher Scientific, Waltham, MA, USA). Assay IDs for molecular probes: Hs00387426_m1—MAP2K4; Hs01547883_g1—MAP2K7; Hs01548508_m1—MAPK8; Hs01558224_m1—MAPK9; Hs02786624_g1—GAPDH; and Hs01060665_g1—ACTB. The reaction was performed on the QuantStudio™5 Real-Time PCR System (Applied Biosystems™, USA) using the reagent kit TaqMan™ Fast Advanced Master Mix (Thermo Fisher Scientific, USA). The following reaction mixture was used for each sample: 10 µL of MasterMix, 1 µL of FAM-labeled molecular probe, 1 µL of VIC-labeled molecular probe, 7 µL of water, and 1 µL of cDNA. Reactions for each sample were conducted in duplicate with a negative control sample used for each series. The relative expression coefficients (R) of the tested genes were determined using the ΔΔCt method.

#### 4.2.4. Bioinformatics Analysis

The gene expression data obtained from cancer tissues were compared with those obtained from normal colon tissues in the Cancer Genome Atlas (TCGA) RNA-seq dataset, available in the UALCAN database (http://ualcan.path.uab.edu/index.html, accessed on 18 April 2025) [22]. The correlation between the expression of the selected genes (*MAPK8*, *MAPK9*, *MAP2K4*, *MAP2K7*) and survival probability for patients with CRC was analyzed using the Kaplan–Meier plotter (https://kmplot.com/analysis/, accessed on 7 October 2025) [23]. The expression of MAP2K4, MAP2K7, MAPK8, and MAPK9 proteins in cancer and normal colon tissue was analyzed using images from the Human Protein Atlas (https://www.proteinatlas.org/, accessed on 12 October 2025) [24]. The association between *MAP2K4*, *MAP2K7*, *MAPK8*, and *MAPK9* gene expression and the presence of mutations in the *KRAS*, *PIK3CA*, *BRAF*, and *TGFB1* genes was assessed using the TIMER 2.0 platform (https://compbio.cn/timer2/, accessed on 11 October 2025) [18]. The analysis of the association between the expression levels of the *MAP2K4*, *MAP2K7*, *MAPK8*, and *MAPK9* genes and the molecular subtype of CMS colorectal cancer was performed using data available at cBIOPortal (https://www.cbioportal.org/, accessed on 20 October 2025) [20].

#### 4.2.5. Statistical Analysis

The statistical analysis was performed using Statistica 13.1 software (TIBCO, Palo Alto, CA, USA), assuming a significance level of *p* = 0.05 for all analyses. The normality of the distribution of the studied variables was checked using the Shapiro–Wilk test. The relative expression coefficients of the studied genes were subjected to Box–Cox transformation before analysis. The correlations between the expression of the studied genes and selected clinicopathological features of the CRC patients were assessed using ANOVA tests and, if necessary, Tukey’s test. Due to the lack of some clinical data for individual patients, some analyses were performed on groups of different sample sizes.

## 5. Conclusions

Our study highlights the critical role of genes in the MAPK pathway in maintaining cellular homeostasis and regulating carcinogenic processes within the large intestine. However, further in-depth investigation of the molecular mechanisms that underpin the initiation and progression of CRC is needed, as this can significantly enhance the discovery of novel biomarkers. Such advancements are instrumental in the early detection and management of CRC, which can ultimately improve prognosis. Furthermore, patient outcomes and survival can be improved by molecules, such as miRNAs, that can modulate *MAPK* gene expression; these appear to be crucial elements in the targeted therapeutic area of CRC. Therefore, these molecular pathways and their regulators may play a pivotal role in the development of novel therapeutic strategies for CRC.

## Figures and Tables

**Table 1:** Comparison of MAP2K4, MAP2K7, MAPK8, and MAPK9 gene expression in relation to the presence or absence of mutations in the KRAS, PIK3CA, BRAF, and TGFB1 genes. *p*-values were calculated using the Wilcoxon test.

*KRAS* Mutant vs. Wild-Type				
Gene Expression Level:	*MAP2K4*	*MAP2K7*	*MAPK8*	*MAPK9*
COAD—colon adenocarcinoma (*n* = 401)	−0.002	0.005	−0.03	0.006
READ—rectum adenocarcinoma (*n* = 144)	−0.058	0.022	−0.072	−0.005
*PIK3CA* Mutant vs. Wild-type				
COAD (*n* = 401)	0.014	−0.027	0.012	−0.016
READ (*n* = 144)	0.059	−0.038	−0.033	0.021
*BRAF* Mutant vs. Wild-type				
COAD (*n* = 401)	0.14 higher level in mutant *p* < 0.05	0.021	0.112 higher level in mutant *p* < 0.05	0.007
READ (*n* = 144)	0.199	0.048	0.106	−0.034
*TGFB1* Mutant vs. Wild-type				
COAD (*n* = 401)	0.171 higher level in mutants *p* < 0.05	0.017	0.064	0.055
READ (*n* = 144)	0.056	−0.03	−0.062	0.245

**Table 2:** Clinical characteristics of the study group.

Characteristic	Categories	N (%)
Tumor location	Rectum	27 (50.9)
	Sigmoid colon	12 (22.6)
	Ascending colon	5 (9.4)
	Recto-sigmoid junction	4 (7.6)
	Splenic flexure	2 (3.8)
	Hepatic flexure	2 (3.8)
	Transverse flexure	1 (1.9)
AJCC stage	I	15 (29.4)
	II	16 (31.4)
	III	14 (27.4)
	IV	6 (11.8)
Tumor size (TNM classification)	Tis	1 (1.9)
	T2	14 (27.5)
	T3	27 (52.9)
	T4	9 (17.7)
Lymph nodes metastases (TNM classification)	N0	31 (60.8)
	N1	10 (19.6)
	N2	10 (19.6)
Distant metastases (TNM classification)	M0	44 (88.0)
	M1	6 (12.0)
Histological grade	Low grade (G1,G2)	39 (73.6)
	High grade (G3,G4)	14 (26.4)
Angioinvasion	Present	22 (43.1)
	Absent	29 (56.9)
Neuroinvasion	Present	12 (23.5)
	Absent	39 (76.5)

## Data Availability

The original contributions presented in this study are included in the article. Further inquiries can be directed to the corresponding author.
